# Integrating Mental Health Screening in Community Pharmacies: Knowledge, Attitudes, and Perceived Barriers Among Pharmacy Students

**DOI:** 10.1155/tswj/9769309

**Published:** 2025-09-15

**Authors:** Amjad H. Bazzari, Firas H. Bazzari

**Affiliations:** ^1^Department of Basic Scientific Sciences, Faculty of Science, Applied Science Private University, Amman, Jordan; ^2^Department of Pharmacy, Faculty of Pharmacy, Jerash University, Jerash, Jordan

**Keywords:** community pharmacy, mental health, mental illness, pharmacy education, pharmacy practice, screening

## Abstract

Mental health disorders remain a significant global burden, and access to timely mental healthcare remains limited. Community pharmacies, given their accessibility to the public, are well-positioned for early mental health screening. Here, we aimed to assess pharmacy students' knowledge, attitudes, and perceived barriers in this regard. The study was conducted via a printed questionnaire, and a total of 211 students, with a mean age of 23.3 ± 3.32 years, participated, including males (35.55%) and females (64.45%). Most students agreed that they are familiar with common mental health disorders (44.08%) and the roles of pharmacists in mental healthcare (38.39%), but were neutral in terms of their ability to identify signs and symptoms (43.13%), awareness of mental health screening tools (32.7%), and confidence in their ability to perform screening (28.44%), with males reporting higher awareness and confidence (*p* < 0.05) than females. In terms of attitudes, the majority strongly agreed that mental healthcare is essential (50.24%), and most agreed that pharmacists are well-positioned for mental health screening (40.28%) and that screening can improve patient outcomes (39.81%), reduce stigma (37.44%), and should be a routine pharmacy practice (30.81%). The overall score of self-perceived knowledge was 59%, while that for positive attitude was 69%. Lastly, the participants moderately agreed on several potential barriers, including time constraints, lack of pharmacist training or education, lack of sufficient privacy, patient reluctance to share and communicate, and stigma toward mental illness among pharmacists, with agreement scores ranging from 53.25% to 64.75%. The results indicate a low level of perceived knowledge, moderately positive attitudes, and relative awareness of the potential barriers, suggesting a need for improved awareness and education in this regard.

## 1. Introduction

Mental illness is a significant global health concern, with an increasing prevalence and economic burden [1]. Numerous measures have been undertaken to increase awareness regarding mental health and illness [2]; however, timely access to mental healthcare is still a major issue in many settings due to several factors, such as a shortage of mental health professionals and stigma toward mental illness [3]. Therefore, multiple initiatives to expand access to mental healthcare have suggested the integration of community pharmacists, given their easy access and frequent interactions with the public, thus presenting a promising, yet underutilized, setting for early screening of mental illness [4].

Pharmacists' roles are increasingly recognized in many settings beyond their basic role of dispensing medications [5]. For instance, pharmacists participate effectively in health promotion, chronic disease management, patient counseling, and medication management review, shifting pharmacists' roles from drug distribution to patient care [6]. In the context of mental healthcare, pharmacists are well-positioned to provide a number of services, such as identifying early signs of mental illness, patient support, and referrals [7], that, if integrated into their daily routine, can possibly bridge the existing gaps to facilitate access to timely interventions. Nevertheless, several interrelated factors may influence the successful implementation, which largely depends on the pharmacists' knowledge regarding mental health, their attitudes, and the existing barriers to such services.

On the other hand, pharmacy students, as future healthcare professionals, will have a crucial role in advancing pharmaceutical services, including mental healthcare. The students' education, experiences, and perceptions will have a direct influence on how these services are delivered. Therefore, assessing and understanding their current level of knowledge regarding mental health, in addition to their attitudes and willingness to be involved in mental healthcare, as well as the perceived barriers, are all vital factors in providing guidance and informing curriculum development, policy-making, and service integration.

This study is aimed at exploring the knowledge, attitudes, and perceived barriers in a university sample of undergraduate pharmacy students regarding the integration of mental health screening into community pharmacy practice. The results of this work will aid in informing educational developments and policy-making to better enhance the preparedness of pharmacy students to align with the evolving demands of healthcare systems and the mental health needs of the population.

## 2. Methods

### 2.1. Sampling and Ethical Considerations

The study was conducted using a printed questionnaire, written in English, and distributed by the researchers among pharmacy students in a Jordanian university. The eligibility criteria included adult age (18 years old and above) and active enrollment status in the pharmacy program, and a total of 211 students participated. Each participant was informed, before receiving the questionnaire, about the study's aims, significance, voluntary participation, data protection, and privacy statement, and that the collected data would be used solely for research purposes, with no identifying information requested. The students were not paid or compensated by any means for their participation, and each one signed an informed consent to take part in the study.

Ethical approval was obtained from the Institutional Review Board at the Deanship of Scientific Research and Postgraduate Studies, Jerash University (Approval Number: 21/5/2024/2025, Date: April 30, 2025). The study also adhered to the guidelines of the Declaration of Helsinki regarding studies involving human subjects [8].

### 2.2. Questionnaire

The questionnaire was initially assessed by three independent experts in the fields of clinical pharmacy and pharmacy practice to ensure content validity. Furthermore, face validation was performed, via a pilot among a small number of the target population, to assess the questionnaire in terms of clarity, comprehension, and relevance. The questionnaire consisted of four main sections: (1) demographics, (2) perceived knowledge, (3) attitudes, and (4) perceived barriers.

The demographics section included five questions: (1) age, (2) gender, (3) year of study (1st, 2nd, 3rd, 4th, or 5th), as pharmacy is a 5-year program offered by Jordanian universities, (4) “have you ever interacted with mental health patients?,” and (5) “have you started your field pharmacy training?,” with the latter two questions having a “Yes” or “No” answer.

The second section was designed to assess the degree of self-perceived knowledge regarding mental healthcare, by the agreement level to five questions: (1) “I am familiar with common mental health disorders such as anxiety, depression, bipolar disorder and schizophrenia.” (2) “I am aware of the role of pharmacists in managing mental health issues, such as providing support, resources, and referrals.” (3) “I am able to identify the signs and symptoms of mental health issues.” (4) “I am aware of the screening tools used for mental health assessment.” (5) “I am confident in my ability to perform mental health screening.”

The attitudes section also included five Likert-like agreement scale questions: (1) “I believe that mental healthcare is essential and a crucial part of overall patient healthcare.” (2) “I believe that pharmacists are well-positioned to screen for mental health issues.” (3) “I believe that mental health screening in pharmacies can improve patient outcomes.” (4) “I believe that screening for mental health in pharmacies will reduce stigma towards mental illness and encourage patients to seek help.” (5) “I believe that mental health screening should be a routine part of pharmacy practice.”

The last section included five other Likert-like agreement scale questions regarding the perceived barriers from the point of view of the students, which were as follows: (1) “There isn't enough time to perform mental health screening.” (2) “There is a lack of pharmacist training or education to perform mental health screening.” (3) “Pharmacies don't offer enough patient privacy to perform mental health screening.” (4) “Most patients would be reluctant to share information or communicate regarding their mental health.” (5) “Stigma among pharmacists towards mental illness and mental health patients would discourage many of them from performing mental health screening.”

Responses to the questions in the second, third, and last sections of the study were recorded using a Likert-like agreement scale: *strongly disagree*, *disagree*, *neutral*, *agree*, and *strongly agree*, with a corresponding score ranging from 0 to 4 (*strongly disagree* = 0; *strongly agree* = 4).

### 2.3. Statistical Analysis

Data was analyzed using JASP software (http://www.jasp-stats.org/). The association between gender and demographic factors was determined using the chi-square test for previous patient interaction and field training variables and the Mann–Whitney *U* test for age and year of study variables, which showed a significant deviation from normal distribution as assessed using the Shapiro–Wilk test. The participant responses to the knowledge, attitude, and barrier questions were coded into ordinal variables, which were used to calculate response scores across questions, and the ranks were compared based on gender, previous patient interaction, and field training using the Mann–Whitney *U* test. The correlation between response ranks and participants' age and year of study was assessed through Spearman's rank correlation test. The effect size was calculated using rank-biserial correlation (rb) for Mann–Whitney *U* tests, the correlation coefficient (rho, *ρ*) for Spearman's correlation tests, and Cramer's *V* for chi-square tests. The internal reliability of the knowledge and attitude questions was assessed using Cronbach's alpha value. All statistical tests were two-tailed at an *α* error of 0.05, and, accordingly, a *p* value less than 0.05 was used to determine significance.

## 3. Results

### 3.1. Participant Demographics

A total of 211 pharmacy students, with a mean age of 23.3 ± 3.32 years, participated including males (*n* = 75, 35.55%) and females (*n* = 136, 64.45%). The sample was inclusive in terms of field training, year of study from 1st to 5th year, and previous interaction with patients suffering from mental health disorders. Most participants have started their field training (*n* = 144, 68.25%) and previously interacted with mental health patients (*n* = 116, 54.98%). No significant difference was observed between males and females for the collected demographics (*p* > 0.05), except for year of study, with higher ranks among males compared to females (*U* = 4237.5, *p* < 0.05). The collected demographic factors are summarized in Table 1.

### 3.2. Perceived Knowledge

The next section of the questionnaire assessed the degree of perceived knowledge regarding mental health and screening through a Likert-like agreement scale, from *strongly disagree* to *strongly agree*. The internal consistency of participants' responses was adequate (Cronbach's *α* = 0.7, 95% CI: 0.63–0.759). Firstly, most participants agreed to being familiar with common mental health disorders (*n* = 93, 44.08%), followed by those who are *neutral* (*n* = 65, 30.81%), *disagree* (*n* = 22, 10.43%), *strongly agree* (*n* = 16, 7.58%), and *strongly disagree* (*n* = 15, 7.11%). The response distribution to this question, by ranks, was independent of all collected participant demographics (*p* > 0.05). Next, the students reported on their awareness of the role of pharmacists in managing mental health issues, and most responded with *agree* (*n* = 81, 38.39%), followed by *strongly agree* (*n* = 52, 24.64%), *neutral* (*n* = 50, 23.7%), *disagree* (*n* = 24, 11.37%), and *strongly disagree* (*n* = 4, 1.9%). The response distribution to the second question was independent of gender, previous patient interaction, and field pharmacy training (*p* > 0.05). However, it negatively correlated with age (*ρ* = −0.139, *p* < 0.05), but not year of study (*p* > 0.05). Then, the students assessed their ability to identify the signs and symptoms of mental health issues, and the majority were *neutral* (*n* = 91, 43.13%), followed by those who *agree* (*n* = 62, 29.38%), *strongly agree* (*n* = 27, 12.8%), *disagree* (*n* = 26, 12.32%), and *strongly disagree* (*n* = 5, 2.37%). The response distribution to this question was independent of all collected demographics (*p* > 0.05). Next, the students were asked regarding their awareness of screening tools used in mental health assessment, and most were *neutral* (*n* = 69, 32.7%), followed by those who *agree* (*n* = 55, 26.07%), *disagree* (*n* = 41, 19.43%), *strongly agree* (*n* = 28, 13.27%), and then *strongly disagree* (*n* = 18, 8.53%). The last question in the knowledge section was to assess perceived confidence in their ability to perform mental health screening, and most participants responded with *neutral* (*n* = 60, 28.44%), followed by *agree* (*n* = 55, 26.07%), *disagree* (*n* = 41, 19.43%), *strongly agree* (*n* = 34, 16.11%), and *strongly disagree* (*n* = 21, 9.95%). The response ranks to both awareness and confidence in screening tools and ability to conduct screening, respectively, showed higher agreement among males than females (*p* < 0.05) and among those who previously interacted with patients suffering from mental health issues (*p* < 0.05). The participant responses to the perceived knowledge questions and the impact of demographic factors are summarized in Table 2.

The response scores of participant responses, representing the level of perceived knowledge, ranged from 2.16 (54%) for awareness of screening tools to 2.73 (68.25%) for awareness of pharmacist roles in mental healthcare. The overall mean score across all knowledge questions was 2.36 ± 0.225 (equating to a score of 59%).

### 3.3. Attitudes

The next section of the questionnaire is aimed at assessing student attitudes toward mental health and the integration of mental health screening in community pharmacies using five Likert-like agreement questions, and the participant responses had a high level of internal consistency (Cronbach's *α* = 0.854, 95% CI: 0.82–0.883). First, the students were asked regarding their belief that mental healthcare is essential and a crucial part of overall healthcare, and the majority *strongly agree* (*n* = 106, 50.24%), followed by those who *agree* (*n* = 58, 27.49%), *neutral* (*n* = 29, 13.74%), *disagree* (*n* = 12, 5.69%), and *strongly disagree* (*n* = 6, 2.84%). The response distribution was independent of gender (*p* > 0.05), but varied across all other demographic factors. Interestingly, the response ranks negatively correlated with age (*ρ* = −0.171, *p* < 0.05) and year of study (*ρ* = −0.257, *p* < 0.01) and were lower (indicating less agreement) among participants who previously interacted with mental health patients (*U* = 6575, *p* < 0.01) and who started their field training (*U* = 5747.5, *p* < 0.05). On the other hand, none of the four remaining attitude questions associated or correlated with any of the collected demographic factors (*p* > 0.05). The second question was about their belief that pharmacists are well-positioned to screen for mental health issues, and most participants responded with *agree* (*n* = 85, 40.28%), followed by *neutral* (*n* = 55, 26.07%), *strongly agree* (*n* = 41, 19.43%), *disagree* (*n* = 21, 9.95%), and *strongly disagree* (*n* = 9, 4.27%). Next, the belief that mental health screening in pharmacies can improve patient outcomes was assessed, and a similar distribution was observed, with most participants responding with *agree* (*n* = 84, 39.81%), followed by *neutral* (*n* = 58, 27.49%), *strongly agree* (*n* = 42, 19.91%), *disagree* (*n* = 18, 8.53%), and *strongly disagree* (*n* = 9, 4.27%). Then, the participants were asked about the belief that mental health screening in pharmacies will reduce stigma toward mental illness and encourage patients to seek help, and most participants responded with *agree* (*n* = 79, 37.44%), followed by *strongly agree* (*n* = 59, 27.96%), *neutral* (*n* = 45, 21.33%), *disagree* (*n* = 19, 9%), and *strongly disagree* (*n* = 9, 4.27%). Lastly, the belief that mental health screening should be a routine part of pharmacy practice was assessed, and most participants *agree* (*n* = 65, 30.81%), followed by those who are either *neutral* (*n* = 56, 26.54%) or *strongly agree* (*n* = 56, 26.54%), then those who *disagree* (*n* = 23, 10.9%), and lastly who *strongly disagree* (*n* = 11, 5.21%). The mean attitude scores, by question, of participant responses ranged from 2.61 (65.25%) for agreement that pharmacists are well-positioned to screen for mental health issues to 3.17 (79.25%) for believing that mental healthcare is essential and a crucial part of overall patient care. The overall mean score across all attitude questions was 2.76 ± 0.237 (equating to a score of 69%), indicating moderately positive attitudes. The distribution, scores, and impact of demographic factors on participant responses to the attitudes' questions are summarized in Table 3.

### 3.4. Perceived Barriers

The last section of the questionnaire is aimed at determining the main barriers to the implementation of mental health screening in pharmacies from the perspective of pharmacy students. A total of five potential barriers were selected and used to formulate five statements, to which the participants would report their agreement level. These included time constraints, lack of pharmacist training or education, privacy concerns, patient reluctance to share or communicate, and stigma toward mental illness by pharmacists. Regarding each barrier, most participants were either in agreement, as with lack of pharmacist training (*n* = 73, 34.6%), lack of enough privacy (*n* = 76, 36.02%), and reluctance of patients to share or communicate (*n* = 81, 38.39%), or neutral as with lack of enough time (*n* = 68, 32.23%) and stigma toward mental illness among pharmacists (*n* = 67, 31.75%). None of the collected demographic factors influenced the response distribution for any of the barriers, with the exception of stigma toward mental illness among pharmacists that would discourage many of them from performing mental health screening. A significant impact for this barrier was observed for year of study, which had a positive correlation (*ρ* = 0.139, *p* < 0.05) and field training (*U* = 3982.5, *p* < 0.05). The results indicate higher agreement ranks with the impact of stigma as a potential barrier to implementing mental health screening in pharmacies among students of higher years and students who started their field training. Similar to score calculation for the knowledge and attitude domains, response scores for each barrier, as indicators of participant agreement level, were calculated and ranged from 2.13 (53.25%) for the lack of enough time to 2.59 (64.75%) for the reluctance of patients to share information or communicate regarding their mental health. The participant responses to the barriers' questions, their scores, and impact of demographic factors are summarized in Table 4.

## 4. Discussion

This study is the first to explore the perceived knowledge, attitudes, and barriers among undergraduate pharmacy students regarding the integration of mental health screening into the daily practice of community pharmacies. The study sample was representative in terms of age and gender distribution and consistent with the data concerning pharmacists and the healthcare system in Jordan, as highlighted by the 2024 annual statistical report by the Jordanian Ministry of Health [9].

Interestingly, younger students reported being more aware of the potential roles of community pharmacists in mental health, which may reflect the current heightened emphasis on mental health or might be influenced by generational differences, where younger generations tend to desire their work to be meaningful and have social impact [10]. However, these are just potential interpretations, and further qualitative research is needed to explore these underlying motivations. Furthermore, more confidence and awareness of the screening tools were reported by participants with previous interaction with mental health patients, suggesting a potential influence of direct exposure to patients with mental illness on preparedness in this regard [11]. Paradoxically, older students and those with field training experience were less likely to consider mental healthcare as an essential part of overall patient care. This might be attributed to a number of possible factors, such as their exposure to real-world limitations in practice and the growing sense of professional boundaries [12, 13]. Moreover, those with field training experience were more likely to suggest stigma among community pharmacists as a barrier, which might be due to encountering such observations or attitudes in practice settings. This aligns with rising evidence noting stigma among healthcare workers as a major barrier to effective mental healthcare [14]. Collectively, these findings highlight the need for further qualitative studies to better understand how demographic and interpersonal factors may influence pharmacists' views on their potential roles in mental healthcare.

With regard to self-reported knowledge, the participants' responses indicate a low level of knowledge and confidence regarding multiple essential aspects of mental health screening, which highlights the need to address this gap. Various approaches have proved to be successful in fostering students' knowledge and competence in educational and clinical settings. For instance, the addition of an elective mental health course to pharmacy curricula has been shown to positively influence the students' perceptions of mental illnesses and improve other aspects related to social distance and stigmatizing behavior toward mental illness [15]. Moreover, O'Reilly et al. have also shown that delivering mental health first aid training for pharmacy students had a significant impact on the participants' ability to correctly identify mental illness, recognize helpful interventions, and boost their confidence in providing pharmaceutical care services for individuals with mental illness [16]. In addition, various simulation-based training sessions, including live direct patient interaction, prerecorded scenarios, and role-plays, have proved to be effective in improving the students' outcomes in terms of mental health knowledge, attitudes, and empathy, which, in turn, highlights their potential in enhancing mental healthcare skills among community pharmacists [17]. Furthermore, continuous professional development (CPD) programs focusing on mental healthcare for new graduates and pharmacists in the workforce also play a major role in enhancing knowledge, attitudes, and, overall, the quality of provided services [18]. All in all, various approaches can be adopted by pharmacy schools and related parties to promote the future role of pharmacists in mental healthcare and establish a framework to govern the practice and ensure that pharmacists are well-equipped with the necessary knowledge.

While positive attitudes were reported regarding the future role of community pharmacists in mental healthcare, an agreement on several potential barriers hindering its implementation was also observed. In addition to low knowledge and lack of proper training, which was discussed earlier, time constraints, privacy, and stigma also pose a challenge. Time constraints are a major issue among community pharmacists that impact not only the quality of patient care but also the pharmacist's well-being [19]. This, according to Al-Diery and Hussain, could be attributed to the “outdated expectations” that “frame pharmacists primarily as medication suppliers,” which requires rethinking both organizational processes and pharmacists' roles [20]. Other studies have suggested the need for additional staff in the pharmacy setting to address this issue [21], and others suggest the proper utilization of artificial intelligence [22], which, collectively, could free up time for effective pharmaceutical care services. Privacy concerns among patients, especially when dealing with mental health–related issues, are another challenge to overcome. A study by Alrasheed et al. has noted the importance of protecting patients' privacy in pharmacy settings and highlighted the need for standards and measures to ensure and improve patient privacy, such as the mandatory availability of a designated counseling space and pharmacists' training to handle privacy-related issues [23]. As for stigma, whether self-stigma among patients or stigmatizing behaviors among pharmacists, it remains a key barrier to overcome. Collaborative efforts on a national scale are needed to spread awareness about mental healthcare among the public and to mitigate any stigmatizing behaviors by healthcare workers.

The current results show certain consistencies and discrepancies regarding mental health knowledge and attitudes, with the results of previous studies on pharmacy students from different regions and populations. A US-based study found that undergraduate pharmacy students have higher stigma levels for mental health treatment and are significantly less likely to seek counseling help compared to medical students (11% and 48%, respectively), suggesting less-positive attitudes compared to the current results [24]. On the other hand, a study conducted on pharmacy students in Ireland found generally positive attitudes toward the provision of mental healthcare and medication optimization to people with mental illness, with around 64% interested in seeking a mental health–related career. However, the study also reported that less than half of students indicated competence and confidence in caring for patients suffering from more severe mental illness [25]. These findings are consistent with the current results estimating 59% and 69% of knowledge and positive attitudes, respectively. Similarly, a study conducted in Saudi Arabia found a positive correlation between mental health literacy and help-seeking among pharmacy students, with a literacy score of 112.53 on the Mental Health Literacy Scale (MHLS), which has a possible score range from 35 to 160 [26], thus equating to a literacy percentage of 62%, which is also quite consistent with the current results [27]. In contrast, another Saudi Arabia–based study reported around 57.3% of negative views toward people with mental illness (score of 60.16 from a possible range of 0–105) and knowledge of mental illness causes of 36.5% (score of 2.92 from a possible range of 0–8), suggesting less positivity and lower knowledge compared to the current study sample [28]. However, caution is required when interpreting different domains of knowledge and when comparing actual versus perceived or self-reported levels of knowledge; for instance, a US-based study assessing the knowledge and confidence of pharmacy students in depression screening showed preintervention scores of 70.7% and 29.3%, respectively [29]. Many factors may be argued to underlie such variation among pharmacy students, which would impact the quality of delivered care, such as communication skills, empathy, and alexithymia [30]. These findings highlight many similarities and also variations regarding mental healthcare knowledge and attitudes among pharmacy students across different cultures and populations. Lastly, variations between perceived barriers to mental healthcare provision and mental health help-seeking could also exist, which may arise from discrepancies between perceived self-stigma and stigma toward others, as observed for doctor of pharmacy (PharmD) students in the United States [31].

Ultimately, the current work acknowledges several limitations for future research. Firstly, the study was conducted using a sample from one Jordanian university, which limits the external validity and generalizability of the findings to other populations. It is important to note that several factors may have influenced the results, for instance, cultural perceptions of mental health in Jordan, including stigma and help-seeking behaviors, which may differ from those in other regions. Additionally, the educational curricula in pharmacy schools in Jordan may also vary internationally, which could influence students' preparedness and attitudes toward mental health services. These factors should be considered when interpreting the findings and their applicability to other settings. Secondly, the study relied on self-reported measures of perceived knowledge rather than incorporating objective assessments. This approach may lead to over- or underestimation of actual competence. In addition, the questionnaire was voluntary and self-administered, which may have introduced response bias, as students with greater interest in mental health or with more favorable attitudes may be more likely to participate. Accordingly, future research should consider using mixed methods and objective assessment tools to better evaluate the actual level of knowledge among participants. Lastly, there is also a need for further longitudinal studies to assess the impact of future interventions on knowledge and attitudes. In addition, qualitative research could offer additional insights into past experiences among students shaping their attitudes and perceptions in this regard.

## 5. Conclusions

The findings of the current work revealed areas for improvement to be considered by pharmacy educators and policy-makers for the successful implementation of mental health screening in community pharmacies. Pharmacy schools should consider curricula development to integrate further theoretical knowledge and more focused training that involves early exposure to patients and interactive exercises to equip the students with the necessary skills and competencies. Additionally, public campaigns and CPD programs for pharmacists could significantly aid in limiting stigma and fostering openness and advocacy for mental health. These initiatives should be paralleled by structural changes that allow pharmacists the time, space, and resources to effectively conduct mental health screenings in a community pharmacy setting.

## Figures and Tables

**Table 1:** Participant demographic factors.

**Variable**	**Total**	**Males**	**Females**	**p** **value** ^**a**^ **(effect size)** ^**b**^
Age, mean (SD)	23.33 (3.32)	23.03 (2.87)	23.5 (3.54)	0.674 (0.035)
Year of study, *n* (%)				0.036^∗^ (0.169)
1st year	10 (4.74%)	4 (5.33%)	6 (4.41%)	
2nd year	32 (15.17%)	8 (10.67%)	24 (17.65%)	
3rd year	64 (30.33%)	18 (24%)	46 (33.82%)	
4th year	48 (22.75%)	19 (25.33%)	29 (21.32%)	
5th year	57 (27.01%)	26 (34.67%)	31 (22.79%)	
Patient interaction, *n* (%)				0.095 (0.115)
No	95 (45.02%)	28 (37.33%)	67 (49.26%)	
Yes	116 (54.98%)	47 (62.67%)	69 (50.74%)	
Field training, *n* (%)				0.384 (0.06)
No	67 (31.75%)	21 (28%)	46 (33.82%)	
Yes	144 (68.25%)	54 (72%)	90 (66.18%)	

^a^Chi-square test, except age and year of study (Mann–Whitney *U* test).

^b^Effect size: Cramer's *V* for chi-square test and rank-biserial correlation for *U* test.

⁣^∗^Significant (*p* < 0.05).

**Table 2:** Perceived knowledge of mental health issues and screening.

**Questions and responses**	**n**	**%**	**Score** ^**a**^ **(0–4)**	**Impact of demographics, ** **p** **value** ^**b**^ **(effect size)** ^**c**^				
				**Gender**	**Age**	**Year of study**	**Patient interaction**	**Field training**
Q1. I am familiar with common mental health disorders such as anxiety, depression, bipolar disorder and schizophrenia								
*Strongly disagree*	15	7.11%	2.35	0.196 (0.101)	0.102 (−0.113)	0.609 (−0.035)	0.397 (0.064)	0.356 (0.074)
*Disagree*	22	10.43%						
*Neutral*	65	30.81%						
*Agree*	93	44.08%						
*Strongly agree*	16	7.58%						
Q2. I am aware of the role of pharmacists in managing mental health issues, such as providing support, resources, and referrals								
*Strongly disagree*	4	1.90%	2.73	0.154 (0.114)	0.044^∗^ (−0.139)	0.421 (−0.056)	0.338 (0.073)	0.775 (0.024)
*Disagree*	24	11.37%						
*Neutral*	50	23.70%						
*Agree*	81	38.39%						
*Strongly agree*	52	24.64%						
Q3. I am able to identify the signs and symptoms of mental health issues								
*Strongly disagree*	5	2.37%	2.38	0.713 (0.029)	0.963 (−0.003)	0.45 (0.052)	0.469 (0.055)	0.585 (0.044)
*Disagree*	26	12.32%						
*Neutral*	91	43.13%						
*Agree*	62	29.38%						
*Strongly agree*	27	12.80%						
Q4. I am aware of the screening tools used for mental health assessment								
*Strongly disagree*	18	8.53%	2.16	0.021^∗^ (0.187)	0.828 (0.015)	0.311 (0.07)	0.01^∗^ (0.2)	0.327 (0.081)
*Disagree*	41	19.43%						
*Neutral*	69	32.70%						
*Agree*	55	26.07%						
*Strongly agree*	28	13.27%						
Q5. I am confident in my ability to perform mental health screening								
*Strongly disagree*	21	9.95%	2.19	0.048^∗^ (0.161)	0.408 (−0.057)	0.694 (0.027)	0.024^∗^ (0.176)	0.334 (0.081)
*Disagree*	41	19.43%						
*Neutral*	60	28.44%						
*Agree*	55	26.07%						
*Strongly agree*	34	16.11%						

^a^The mean score of responses for each question (*strongly disagree* = 0 and *strongly agree* = 4).

^b^Mann–Whitney *U* test, except age and year of study (Spearman's rank correlation).

^c^Effect size: rank-biserial correlation for *U* test and correlation coefficient for Spearman's correlation.

⁣^∗^Significant (*p* < 0.05).

**Table 3:** Attitudes toward mental healthcare and screening in pharmacies.

**Questions and responses**	**n**	**%**	**Score** ^**a**^ **(0–4)**	**Impact of demographics, ** **p** **value** ^**b**^ **(effect size)** ^**c**^				
				**Gender**	**Age**	**Year of study**	**Patient interaction**	**Field training**
Q1. I believe that mental healthcare is essential and a crucial part of overall patient healthcare								
*Strongly disagree*	6	2.84%	3.17	0.72 (0.028)	0.013^∗^ (−0.171)	<0.01^∗^ (−0.257)	<0.01^∗^ (0.193)	0.015^∗^ (0.191)
*Disagree*	12	5.69%						
*Neutral*	29	13.74%						
*Agree*	58	27.49%						
*Strongly agree*	106	50.24%						
Q2. I believe that pharmacists are well-positioned to screen for mental health issues								
*Strongly disagree*	9	4.27%	2.61	0.547 (0.048)	0.128 (−0.105)	0.092 (−0.116)	0.315 (0.077)	0.076 (0.145)
*Disagree*	21	9.95%						
*Neutral*	55	26.07%						
*Agree*	85	40.28%						
*Strongly agree*	41	19.43%						
Q3. I believe that mental health screening in pharmacies can improve patient outcomes								
*Strongly disagree*	9	4.27%	2.63	0.257 (0.09)	0.281 (−0.075)	0.463 (−0.051)	0.353 (0.071)	0.56 (0.048)
*Disagree*	18	8.53%						
*Neutral*	58	27.49%						
*Agree*	84	39.81%						
*Strongly agree*	42	19.91%						
Q4. I believe that screening for mental health in pharmacies will reduce stigma towards mental illness and encourage patients to seek help								
*Strongly disagree*	9	4.27%	2.76	0.611 (0.041)	0.127 (−0.105)	0.231 (−0.083)	0.601 (0.04)	0.17 (0.113)
*Disagree*	19	9.00%						
*Neutral*	45	21.33%						
*Agree*	79	37.44%						
*Strongly agree*	59	27.96%						
Q5. I believe that mental health screening should be a routine part of pharmacy practice								
*Strongly disagree*	11	5.21%	2.63	0.284 (0.086)	0.419 (−0.056)	0.569 (−0.039)	0.566 (0.044)	0.465 (0.061)
*Disagree*	23	10.90%						
*Neutral*	56	26.54%						
*Agree*	65	30.81%						
*Strongly agree*	56	26.54%						

^a^The mean score of responses for each question (*strongly disagree* = 0 and *strongly agree* = 4).

^b^Mann–Whitney *U* test, except age and year of study (Spearman's rank correlation).

^c^Effect size: rank-biserial correlation for *U* test and correlation coefficient for Spearman's correlation.

⁣^∗^Significant (*p* < 0.05).

**Table 4:** Barriers toward implementing mental health screening in pharmacies.

**Questions and responses**	**n**	**%**	**Score** ^**a**^ **(0–4)**	**Impact of demographics, ** **p** **value** ^**b**^ **(effect size)** ^**c**^				
				**Gender**	**Age**	**Year of study**	**Patient interaction**	**Field training**
Q1. There is not enough time to perform mental health screening								
*Strongly disagree*	21	9.95%	2.13	0.688 (0.032)	0.45 (0.052)	0.524 (−0.044)	0.135 (0.116)	0.535 (0.052)
*Disagree*	40	18.96%						
*Neutral*	68	32.23%						
*Agree*	54	25.59%						
*Strongly agree*	28	13.27%						
Q2. There is a lack of pharmacist training or education to perform mental health screening								
*Strongly disagree*	14	6.64%	2.57	0.799 (0.021)	0.401 (−0.058)	0.786 (−0.019)	0.845 (0.015)	0.274 (0.09)
*Disagree*	24	11.37%						
*Neutral*	50	23.70%						
*Agree*	73	34.60%						
*Strongly agree*	50	23.70%						
Q3. Pharmacies do not offer enough patient privacy to perform mental health screening								
*Strongly disagree*	14	6.64%	2.5	0.791 (0.021)	0.953 (−0.004)	0.575 (0.039)	0.893 (0.01)	0.092 (0.139)
*Disagree*	30	14.22%						
*Neutral*	47	22.27%						
*Agree*	76	36.02%						
*Strongly agree*	44	20.85%						
Q4. Most patients would be reluctant to share information or communicate regarding their mental health								
*Strongly disagree*	12	5.69%	2.59	0.276 (0.087)	0.063 (−0.128)	0.85 (−0.013)	0.893 (0.01)	0.732 (0.028)
*Disagree*	17	8.06%						
*Neutral*	59	27.96%						
*Agree*	81	38.39%						
*Strongly agree*	42	19.91%						
Q5. Stigma among pharmacists towards mental illness and mental health patients would discourage many of them from performing mental health screening								
*Strongly disagree*	24	11.37%	2.21	0.805 (0.02)	0.943 (0.005)	0.044^∗^ (0.139)	0.933 (0.007)	0.036^∗^ (0.174)
*Disagree*	31	14.69%						
*Neutral*	67	31.75%						
*Agree*	54	25.59%						
*Strongly agree*	35	16.59%						

^a^The mean score of responses for each question (*strongly disagree* = 0 and *strongly agree* = 4).

^b^Mann–Whitney *U* test, except age and year of study (Spearman's rank correlation).

^c^Effect size: rank-biserial correlation for *U* test and correlation coefficient for Spearman's correlation.

⁣^∗^Significant (*p* < 0.05).

## Data Availability

The data that support the findings of this study are available from the corresponding author upon reasonable request.

## References

[1] Arias D., Saxena S., Verguet S. (2022). Quantifying the Global Burden of Mental Disorders and Their Economic Value. *EClinicalMedicine*.

[2] Foulkes L., Andrews J. L. (2023). Are Mental Health Awareness Efforts Contributing to the Rise in Reported Mental Health Problems? A Call to Test the Prevalence Inflation Hypothesis. *New Ideas in Psychology*.

[3] Bazzari A. H., Bazzari F. H. (2023). Assessing Stigma Towards Mental Illness in Relation to Demographics Attitudes and Past Experiences Among Pharmacy Students in a Jordanian University Sample. *Behavioral Sciences*.

[4] Rubio-Valera M., Chen T. F., O’Reilly C. L. (2014). New Roles for Pharmacists in Community Mental Health Care: A Narrative Review. *International Journal of Environmental Research and Public Health*.

[5] Bazzari F. H., Bazzari A. H. (2023). Attitudes and Knowledge Regarding the Therapeutic Use of Cannabinoids Among Community Pharmacists: A Pilot Cross-Sectional Study in Amman, Jordan. *Healthcare*.

[6] Schindel T. J., Yuksel N., Breault R., Daniels J., Varnhagen S., Hughes C. A. (2017). Perceptions of Pharmacists’ Roles in the Era of Expanding Scopes of Practice. *Research in Social and Administrative Pharmacy*.

[7] El-Den S., Collins J. C., Chen T. F., O’Reilly C. L. (2021). Pharmacists’ Roles in Mental Healthcare: Past, Present and Future. *Pharmacy Practice*.

[8] World Medical Association WMA Declaration of Helsinki—Ethical Principles for Medical Research Involving Human Subjects. http://www.wma.net/policies-post/wma-declaration-of-helsinki-ethical-principles-for-medical-research-involving-human-subjects/.

[9] Jordanian Ministry of Health 2024 Annual Statistical Report. https://www.moh.gov.jo/ebv4.0/root_storage/ar/eb_list_page/%25D8%25A7%25D9%2584%25D8%25AA%25D9%2582%25D8%25B1%25D9%258A%25D8%25B1_%25D8%25A7%25D9%2584%25D8%25A7%25D8%25AD%25D8%25B5%25D8%25A7%25D8%25A6%25D9%258A_%25D8%25A7%25D9%2584%25D8%25B3%25D9%2586%25D9%2588%25D9%258A-2024_(1).pdf.

[10] Twenge J. M., Campbell S. M., Hoffman B. J., Lance C. E. (2010). Generational Differences in Work Values: Leisure and Extrinsic Values Increasing, Social and Intrinsic Values Decreasing. *Journal of Management*.

[11] Dornan T., Boshuizen H., King N., Scherpbier A. (2007). Experience-Based Learning: A Model Linking the Processes and Outcomes of Medical Students’ Workplace Learning. *Medical Education*.

[12] Murphy A. L., Phelan H., Haslam S., Martin-Misener R., Kutcher S. P., Gardner D. M. (2016). Community Pharmacists’ Experiences in Mental Illness and Addictions Care: A Qualitative Study. *Substance Abuse Treatment, Prevention, and Policy*.

[13] Alshorman D. Z., Bazzari A. H., Bazzari F. H. (2024). Comprehensive Assessment of Demographic and Occupational Factors Influencing Burnout Amongst Community Pharmacists in Jordan. *Pharmacia*.

[14] Lien Y. Y., Lin H. S., Lien Y. J. (2021). Challenging Mental Illness Stigma in Healthcare Professionals and Students: A Systematic Review and Network Meta-Analysis. *Psychology & Health*.

[15] Gable K. N., Muhlstadt K. L., Celio M. A. (2011). A Mental Health Elective to Improve Pharmacy Students’ Perspectives on Mental Illness. *American Journal of Pharmaceutical Education*.

[16] O’Reilly C. L., Bell J. S., Kelly P. J., Chen T. F. (2011). Impact of Mental Health First Aid Training on Pharmacy Students’ Knowledge, Attitudes and Self-Reported Behaviour: A Controlled Trial. *Australian & New Zealand Journal of Psychiatry*.

[17] Ung T. X., El-Den S., Moles R. J., O’Reilly C. L. (2023). The Use of Mental Health Simulation in Pharmacy Practice and Education: A Systematic Review. *American Journal of Pharmaceutical Education*.

[18] Wheeler A., Mey A., Kelly F., Hattingh L., Davey A. K. (2014). Education and Training for Community Pharmacists in Mental Health Practice: How to Equip This Workforce for the Future. *Journal of Mental Health Training, Education and Practice*.

[19] Bou-Saba A. W., Kassak K. M., Salameh P. R. (2022). The Current Trends and Challenges Towards Good Community Pharmacy Practice and the Way Forward. *Exploratory Research in Clinical and Social Pharmacy*.

[20] Al-Diery T., Hussain F. N. (2025). Person-Centred Care in Pharmacy: Time to Walk the Talk. *Journal of Pharmaceutical Policy and Practice*.

[21] Karia A., Norman R., Robinson S. (2022). Pharmacist’s Time Spent: Space for Pharmacy-Based Interventions and Consultation Time (SPICE)—An Observational Time and Motion Study. *BMJ Open*.

[22] Bazzari A. H., Bazzari F. H. (2024). Assessing the Ability of GPT-4o to Visually Recognize Medications and Provide Patient Education. *Scientific Reports*.

[23] Alrasheed M. A., Alfageh B. H., Almohammed O. A. (2024). Privacy in Community Pharmacies in Saudi Arabia: A Cross-Sectional Study. *Healthcare*.

[24] Fischbein R., Bonfine N. (2019). Pharmacy and Medical Students’ Mental Health Symptoms, Experiences, Attitudes and Help-Seeking Behaviors. *American Journal of Pharmaceutical Education*.

[25] Keating D., McWilliams S., Clarke M., Strawbridge J. (2023). Pharmacy Student Attitudes to Mental Illness and the Provision of Mental Health Care: A Repeated Cross-Sectional Survey. *International Journal of Clinical Pharmacy*.

[26] O’Connor M., Casey L. (2015). The Mental Health Literacy Scale (MHLS): A New Scale-Based Measure of Mental Health Literacy. *Psychiatry Research*.

[27] Almanasef M. (2021). Mental Health Literacy and Help-Seeking Behaviours Among Undergraduate Pharmacy Students in Abha, Saudi Arabia. *Risk Management and Healthcare Policy*.

[28] Alsahali S. (2021). Knowledge and Attitude of Pharmacy Students Toward People With Mental Illnesses and Help-Seeking: A Cross-Sectional Study From Saudi Arabia. *Pharmacy*.

[29] Habba S., Frond D., Burghardt K., Stewart B. (2025). Navigating Mental Health Conversations: Student Pharmacists Complete Mental Health First Aid Training and Apply It Through Experiential Learning in a Community Pharmacy Depression Screening Program. *INNOVATIONS in Pharmacy*.

[30] Bazzari A. H., Bazzari F. H. (2025). The Correlation Between Alexithymia and Communication Skills Among Undergraduate Pharmacy Students. *Heliyon*.

[31] Buige A., Nguyen M., Harris S. C. (2021). Barriers to Mental Health Care and Stigma Perception in Doctor of Pharmacy Students Across the United States. *Currents in Pharmacy Teaching and Learning*.

